# Comparing Measures of Late HIV Diagnosis in Washington State

**DOI:** 10.1155/2012/182672

**Published:** 2011-11-15

**Authors:** Laura Saganic, Jason Carr, Rosa Solorio, Maria Courogen, Tom Jaenicke, Ann Duerr

**Affiliations:** ^1^Department of Health Services and Epidemiology, University of Washington, Seattle, WA 98195, USA; ^2^Infectious Disease Assessment Unit, Washington State Department of Health, Olympia, WA 98504, USA

## Abstract

As more US HIV surveillance programs routinely use late HIV diagnosis to monitor and characterize HIV testing patterns, there is an increasing need to standardize how late HIV diagnosis is measured. In this study, we compared two measures of late HIV diagnosis, one based on time between HIV and AIDS, the other based on initial CD4^+^ results. Using data from Washington's HIV/AIDS Reporting System, we used multivariate logistic regression to identify predictors of late HIV diagnosis. We also conducted tests for trend to determine whether the proportion of cases diagnosed late has changed over time. Both measures lead us to similar conclusions about late HIV diagnosis, suggesting that being male, older, foreign-born, or heterosexual increase the likelihood of late HIV diagnosis. Our findings reaffirm the validity of a time-based definition of late HIV diagnosis, while at the same time demonstrating the potential value of a lab-based measure.

## 1. Background

Approximately one in five people living with HIV in the United States is unaware of their HIV status [1]. Research suggests that many of these individuals—at least a quarter of a million people—regularly receive health care services, yet they are not tested for HIV [2]. These missed opportunities are costly, preventing early detection of HIV infection and prolonging the HIV epidemic within our nation [3]. The National HIV/AIDS Strategy includes a goal to reduce HIV infections by increasing the proportion of infected individuals who know their status, from an estimated 79% to 90% by 2015 [4]. Accomplishing this goal will require a substantial increase in HIV testing. Moreover, prevention programs will need better ways to identify and characterize people who are at risk for HIV but who are not routinely tested for HIV.

Routine HIV screening, which leads to early diagnosis, is an efficacious and cost-effective strategy for HIV prevention [5–7]. Early diagnosis of HIV infection can reduce the costs of HIV treatment, improve health outcomes, and prevent others from becoming exposed to the virus [8–11]. Since 2006, the U.S. Centers for Disease Control and Prevention (CDC) have recommended that all adult and adolescent patients in health care settings be regularly screened for HIV, regardless of known risk behaviors [2]. Similar recommendations have been issued by the World Health Organization and by the American College of Physicians [12]. Patients and health care providers are encouraged to consider routine HIV screening as a standard medical practice, similar to screenings performed for other chronic health conditions, such as cancer and cardiovascular disease. 

A growing number of US HIV surveillance programs are routinely monitoring late HIV diagnosis, or the proportion of new HIV cases that are diagnosed late in the course of their HIV illness. Late HIV diagnosis is a measure of program performance within the Washington State Department of Health's HIV Prevention Program and is currently one of three HIV-related metrics which are being used by CDC's Winnable Battles effort to monitor and support state-specific progress towards curbing the HIV epidemic [13]. Surveillance data describing late HIV diagnosis provide a measure of HIV testing frequency and help characterize HIV-infected people who are unaware of their HIV status. Yet, there is currently a lack of consensus regarding how late diagnosis should be measured. More than 20 different measures of late diagnosis have been cited in various publications [14]. In 2009, the European Late Presenter Consensus working group established a harmonized definition of late HIV diagnosis [15]. However, in the US, a standard definition has yet to be adopted. Most surveillance programs use a time-based approach in which newly diagnosed HIV cases defined as late are individuals diagnosed with AIDS within a short-time period after initial diagnosis of HIV infection, for example one month [16], three months [17], twelve months [18–20], or even 3 years [21]. However, this approach can require a lengthy follow-up period and hinges on our somewhat limited ability to determine when a diagnosis first occurred. Also, the chosen time interval between diagnosis of HIV and AIDS often varies across jurisdictions. The inconsistencies in defining late HIV diagnosis make examination of factors associated with late diagnosis difficult [17].

Outside the United States, surveillance programs commonly use initial CD4^+^ T-cell count to determine a patient's stage of HIV illness at the time of diagnosis [22]. This approach is not dependent on long-term follow-up and could provide a more reliable and comparable definition for late diagnosis of HIV infection. Yet, while the completeness of laboratory data has improved over time, many US jurisdictions remain wary of potential bias associated with the incomplete reporting of laboratory results [17]. This is also a concern in Washington state, where comprehensive HIV laboratory reporting has only been in place since 2006. 

We designed this study with three objectives in mind. First, we wish to inform state and local HIV prevention efforts by characterizing people with HIV who are diagnosed late in the course of their HIV illness. Second, we wish to determine whether the proportion of new HIV cases diagnosed late has changed over the past decade. Third, we compare two alternative measures of late HIV diagnosis, evaluating whether these measures lead us to similar or different conclusions about late HIV diagnosis in our state, and whether stated concerns about bias associated with either measure are justified. 

## 2. Methods

We used surveillance data from Washington state's core HIV/AIDS reporting system (eHARS). This data system contains information about all individuals who have received a confidential diagnosis of HIV or AIDS while residing in Washington. The state also maintains a comprehensive laboratory reporting system which can be linked to eHARS and which contains all reported CD4^+^ T-cell test results associated with each HIV/AIDS case. 

We analyzed adult cases, ages 18 years and older, who were diagnosed with HIV infection while residing in Washington state between 2000 and 2009. The time-based measure defines a case as late if the individual is diagnosed with AIDS within 12 months of initial HIV diagnosis. In our analysis of the time-based measure, we excluded cases with incomplete or missing dates of HIV or AIDS diagnosis (missing either month or year). When calculating the time-based measure, we also excluded cases diagnosed with HIV in 2009, since reporting delays would prevent us from being able to identify all cases that received an AIDS diagnosis within the 12-month follow-up period. The lab-based measure defines cases as late if the initial CD4^+^ T-cell count is <350 cells/mL, based on current WHO recommendations for HIV treatment initiation. We excluded cases from our analysis if the initial lab result was based on a specimen collected ≥90 days after HIV diagnosis. 

We used SAS software (version 9.2) to generate descriptive statistics and conduct logistic regression, with relative likelihood for late HIV diagnosis described using adjusted odds ratios. Covariates in the multivariate regression model included gender, age at HIV diagnosis, race and Hispanic ethnicity, mode of HIV exposure, county of residence (at diagnosis), and foreign-born status. 

To test for trends over time, we used a JoinPoint regression program (version 3.0) developed by the National Cancer Institute. Slope was calculated based on the model ln⁡(*y*) = *xb*. Standard error of the dependent variable was based on the assumption that the underlying data fit a Poisson distribution. Annual percent change (APC) was used to describe change in the proportion of cases diagnosed late over time. APC assumes that rate of change occurs as a constant percentage over a defined time period. 

## 3. Results

Among the 5,639 new HIV cases in Washington state between 2000 and 2009, 91% had adequate data to calculate a time-based measure of late HIV diagnosis (Table 1). All but one of the cases with incomplete data were diagnosed in 2009, which was too recent to determine whether an AIDS diagnosis took place during the 12-month follow-up period. Over the same time period, 71% of new cases had documentation of a valid CD4^+^ T-cell test result within 90 days of HIV diagnosis. While the proportion of cases without a CD4^+^ T-cell laboratory result was relatively high (29%), it appeared to decrease over time, from 35% in 2000 to only 17% in 2009. Regardless of measure, cases with complete data generally resembled those with missing or incomplete data. There were no statistical differences by gender or race/ethnicity. However, we did observe small but statistically significant differences with respect to age at HIV diagnosis, mode of HIV exposure, and county of residence (lab-based measure only). 

Overall, a lower proportion of new HIV cases was diagnosed late using the time-based measure (37%) compared with the lab-based measure (56%; Table 2). However, within demographic and risk strata, the adjusted odds of being a late HIV diagnosis were similar regardless of measure. Men in our sample had 1.9–2.5 times the odds of being diagnosed late compared to women. We also found strong evidence for a positive association between late diagnosis and increasing age at HIV diagnosis. For example, older adults (ages 45 and older) had 1.8–2.2 times greater odds of being diagnosed late than did adults in their late twenties and early thirties. New HIV cases reporting heterosexual exposure had more than twice the odds of late diagnosis compared to those categorized as men who have sex with men (MSM). Non-MSM male cases, including those with no identified risk category, actually had among the highest proportions of late diagnoses: 54% and 75% according to the time-based and lab-based measures, respectively. Cases residing outside King County at the time of HIV diagnosis had odds of late diagnosis that were nearly 1.4 times larger than cases residing inside King County. 

Foreign-born status was strongly associated with late HIV diagnosis. Overall, the odds of late diagnosis were about 1.3 times greater among cases born outside the United States versus those born within. However, foreign-born status seemed to confound the association between race/ethnicity and late HIV diagnosis (Table 3). Among cases born in the US, there was generally little evidence to suggest an association between race/ethnicity and late HIV diagnosis. The odds of late diagnosis among US-born American Indians and Alaska Natives (AI/AN) were about 1.7 times larger than those among US-born whites. Using the lab-based measure, US-born Hispanics had 1.5 times greater odds of late diagnosis compared to their white counterparts. However, the time-based measure provided no evidence for such an association. Among cases born outside the US, differences in the odds of late diagnosis were much greater between racial/ethnic groups. Among foreign-born cases, nonwhite cases had 1.7–3.4 times greater odds of late diagnosis compared to white cases.

**Figure 1:**
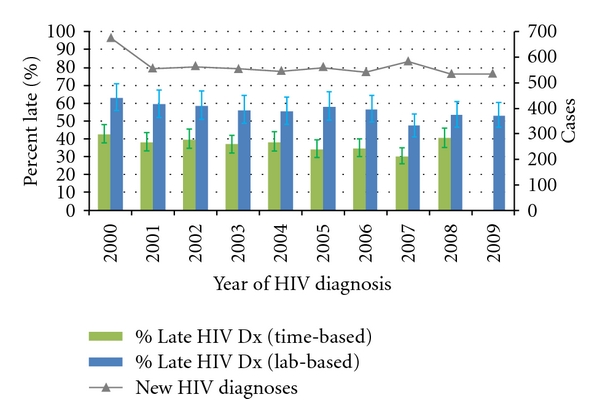
New HIV diagnoses and proportions that were diagnosed late, by year of HIV diagnosis, Washington State, 2000–2009.

**Figure 2:**
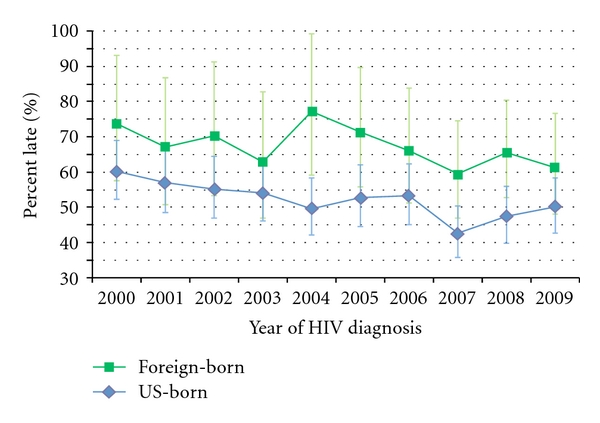
Trends in late HIV diagnosis (lab-based) and foreign-born status, Washington State, 2000–2009.

Statewide, there is some evidence to suggest that the occurrence of late HIV diagnosis has decreased over the past decade (Figure 1). JoinPoint regression of both measures showed an average decrease of about 2 percent per year. However, only the slope associated with the lab-based measure (APC = −2.00) was statistically significant at the *P* = 0.05 level. Most of the change appears to be explained by significant decreases in late diagnosis among US-born cases (APC = −2.53), which comprise roughly 75% of all new HIV cases in Washington (Figure 2). The proportion of new HIV cases that are foreign-born has steadily risen over the past decade.

## 4. Discussion

As our state's HIV epidemic nears the end of its third decade, the proportion of new HIV cases which are diagnosed late remains unacceptably high. Although CDC recommendations for the expansion of HIV testing have been in place for more than five years, a substantial proportion of new cases is still being detected late in the course of their HIV illness, after the point at which treatment should have been initiated. Statewide, declines in late HIV diagnosis over the past ten years appear to be minimal. Indeed, our findings support the notion that targeted HIV testing efforts, which depend heavily on patient and provider perceptions of HIV risk, cannot by themselves reduce the number of HIV-infected people who are infected but unaware of their status [3, 23]. Many of the characteristics we observed to be associated with late HIV diagnosis, such as being heterosexual, or residing in a rural area, are not traditionally considered strong indicators of HIV risk. Therefore, HIV testing efforts need to be broadened to include people who are at elevated risk for HIV but who are, for a variety of reasons, not getting tested.

Men in our sample were more likely to be diagnosed late than women. This finding is consistent with several published studies [24–26]. However, other attempts to characterize the association between gender and late diagnosis have proven inconclusive [18, 27]. Using either measure of late diagnosis, the difference in the proportions of male and female cases that were diagnosed late was relatively small (<2%), suggesting the direction of the gender association could be sensitive to relatively minor differences in testing patterns. As reported elsewhere, we observed men with mode of transmission categorized as MSM or MSM/IDU to be among the least likely to be a late HIV diagnosis [28]. These cases comprise the majority of HIV cases in Washington state. However, since these risk categories do not by definition contain female cases, they were effectively ignored by our multivariate regression model, with gender comparisons and resulting odds ratios limited mainly to cases falling into three risk categories: heterosexuals, injection drug users, and cases with no identified risk factors. 

Although women are generally less likely to perceive themselves as being at risk for HIV, which would seem to favor late diagnosis, they typically demonstrate higher utilization of health services compared to men, and HIV testing is more widely accessible to women as result of both prenatal HIV screening as well as cervical cancer screening [29, 30]. On the other hand, the association between gender and late diagnosis could also be influenced by lower levels of HIV testing within certain male subgroups, some of whom may actually be MSM but have not been reported as such. For example, research suggests that male Latinos at risk for HIV tend to get tested for HIV less frequently, and often do not identify as being gay or bisexual despite engaging in MSM behaviors [31–35].

We expected a positive correlation between increasing age and late diagnosis. The very act of delaying testing requires the passage of time, and as time passes, people get older. Also, as the body ages, CD4^+^ T-cell counts tend to naturally decrease [36]. This results in a less-effective specific immune response, leading to greater viremia and less time to AIDS among individuals who seroconvert [37, 38]. Finally, some research indicates that older MSM test less often than younger MSM [39].

Overall, we expected a stronger relationship between race/ethnicity and late diagnosis than we observed, given the widely documented lower testing rates among racial/ethnic minorities [16, 21]. While crude associations were apparent, racial/ethnic differences in late diagnosis were substantially smaller once we controlled for foreign-born status. The increased risk for late diagnosis among Native Americans is consistent with other evidence showing higher levels of poverty and limited access to health services within this population [40]. Likewise, late diagnosis among Hispanics could be due to barriers to HIV testing, particularly stigma-induced fear of testing positive and difficulty communicating with providers among individuals who do not speak English [41, 42].

Our finding that foreign-born status confounds the associations between race/ethnicity and late HIV diagnosis is consistent with other published findings [2, 18, 43]. While foreign-born cases are more likely than US-born cases to be diagnosed late, it is difficult to determine how much of this increased likelihood is due to lower HIV testing versus other reasons. As result of strict HIV surveillance reporting requirements in the US, some foreign-born cases could be diagnosed outside the US but lack required documentation to demonstrate that a previous diagnosis took place. This could result in misclassification bias, causing some foreign-born cases to appear as late diagnoses when they really are not. Needs assessments data have suggested that as many as one quarter of foreign-born HIV cases diagnosed in our state were actually diagnosed at least one year earlier than the reported date of HIV diagnosis [32, 44]. Yet, differences in testing behaviors could also be explained by other factors associated with recent immigration to the US, such as poor access to HIV testing services, language barriers, social isolation, financial instability, or lack of knowledge about HIV [16, 27, 45].

### 4.1. Data Completeness and Limitations

Differences between cases with and without supporting data were relatively small and similar between late measures. The similarity in findings suggests that both measures would be prone to the same kinds of minimal bias. 

The completeness of data supporting the time-based measure is higher than that of the lab-based measure in our study. However, this is heavily dependent on the duration of the chosen observation period. Had we evaluated late HIV diagnosis over a five-year time period, completeness would have been lower for the time-based definition.

Although we tend to assume that people with AIDS have serious health conditions that compel them to seek treatment, not all cases who develop AIDS seek care in a timely manner. Thus we may have misclassified some cases categorized as “not late” because although they have progressed to AIDS, they have not yet been linked to care or reported to our surveillance system. The time-based measure is also a subject to reporting delays associated with the diagnosis of HIV and AIDS, as well as our ability to monitor diagnoses that take place either out-of-state or outside the country. 

Completeness of the data for the lab-based measure is strongly dependent on whether a given jurisdiction has implemented comprehensive lab reporting. Nevertheless, we are aware that a growing proportion of providers in our state are using out-of-state labs to process HIV-related specimens. Laws governing the reporting of laboratory results vary by state [46]. Although Washington has laws intended to ensure the comprehensive reporting of all HIV and AIDS test results, these laws do not apply to laboratories located outside state borders. Hence, many CD4^+^ T-cell results likely remain unreported each year, especially if they do not correspond to an HIV or AIDS case definition, such as CD4^+^ T-cell counts over 200.

The logistic regression models were based on cross-sectional comparisons and do not indicate whether testing patterns have changed over time. Cases with complete data necessary to calculate either measure of lateness might not be representative of all new HIV cases, resulting in selection bias. Moderate case counts resulted in lower stratified cell sizes and wider confidence intervals that prevented our ability to detect trends in late diagnosis within stratified sub-groups. 

The adjusted odds ratios generated by our logistic regression model provide an indication of which variables are associated with late HIV diagnosis in our state, as well as some idea as to relative strength of those associations. However, since late HIV diagnosis is a common event within our sample, the odds ratios likely represent a substantial overestimation of corresponding relative risks.

Among new HIV cases categorized as foreign-born in our analysis, approximately 20% are missing information about country of birth. Although some of these cases might actually have been born in the US, we do not consider this to be a large limitation. By misclassifying some native-born cases as foreign-born, we would essentially dilute the latter group, making them appear more like native-born cases than they actually are. Accurate classification of these cases would likely result in better separation between native and foreign-born cases, which would likely improve our ability to measure an association and strengthen our findings.

### 4.2. Strengths and Future Implications

To our knowledge, this is the first study which uses HIV surveillance data to compare two measures of late HIV diagnosis. Our results indicate that both measures point to the same risk factors for late HIV diagnosis. The relative strength and direction of these associations were also very similar. 

The lab-based measure is more clinically relevant because it is based on the current recommendations for treatment initiation and can be easily modified as the standard of care evolves. In addition, it allows the inclusion of at least one additional year of data (the most recent). As our laboratory reporting system matures, and with the expected introduction of health information exchanges, the quality and completeness of laboratory data should continue to improve over time [47]. Among the 536 cases with missing data needed to calculate the time-based measure, nearly half were identified as late using the lab-based measure. On the other hand, using both measures together could provide a broader and potentially more informative understanding of late diagnosis, offering additional information about cases that lack adequate data to support either one measure or the other.

Strengths of our study include the use of nine years of statewide surveillance data as well as an evaluation of two different ways of measuring late HIV diagnosis. Also, our use of multiple logistic regression allowed us to control for numerous potential confounders. Our results reaffirm the validity of a time-based definition of late HIV diagnosis, while at the same time demonstrating the potential value of a lab-based measure. Moreover, because it is a subject to fewer potential limitations, the lab-based measure might be a better alternative in jurisdictions with comprehensive laboratory reporting.

## Figures and Tables

**Table 1:** Comparing cases meeting two definitions of late HIV diagnosis, 2000–2009.

Lab-based measure of late diagnosis	Time-based measure of late diagnosis			
	Late	Not late	Missing	Total
	No. (% of total)	No. (% of total)	No. (% of total)	No. (% of total)
Late	1575 (28)	432 (8)	236 (4)	2243 (40)
Not late	113 (2)	1450 (26)	209 (4)	1772 (31)
Missing	216 (4)	1317 (23)	91 (2)	1624 (29)

Total	1904 (34)	3199 (57)	536 (10)	5639 (100)

**Table 2:** Characteristics of late HIV diagnoses, including adjusted* odds ratios, Washington state, 2000–2009.

	Time-based late measure			Lab-based late measure		
	2000–2008			2000–2009		
	(Late = AIDS within 12 months)			(Late = CD4^+^ T-cell < 350 cells/mL)		
	No.	% Late	Odds ratio (95% CI)*	No.	% Late	Odds ratio (95% CI)*
Total late HIV diagnoses	1904	37%	n/a n/a	2243	56%	n/a n/a
Gender						
Male	1622	38%	**1.88 (1.53–2.31)**	1905	56%	**2.54 (1.98–3.25) **
Female	282	36%	Reference	338	54%	Reference
Race/ethnicity						
White, NH	1077	34%	Reference	1273	51%	Reference
Black, NH	382	42%	**1.20 (1.01–1.43)**	423	61%	1.21 (0.98–1.48)
Hispanic	273	43%	**1.33 (1.07–1.65)**	348	66%	**1.61 (1.26–2.05) **
Asian	80	44%	1.35 (0.95–1.90)	102	65%	1.34 (0.92–1.95)
NHOPI**	11	46%	1.68 (0.71–3.95)	11	55%	1.08 (0.43–2.71)
AI/AN	45	48%	**1.68 (1.09–2.60)**	44	67%	**1.94 (1.13–3.34) **
Multiple/unknown	36	39%	1.45 (0.93–2.28)	42	61%	**1.72 (1.03–2.87) **
Age at HIV diagnosis						
18–25 yrs	91	16%	**0.42 (0.33–0.54)**	150	38%	**0.57 (0.45–0.72) **
25–34 yrs	479	30%	Reference	601	51%	Reference
35–44 yrs	753	41%	**1.59 (1.38–1.83)**	847	59%	**1.38 (1.18–1.62) **
45 yrs and over	581	50%	**2.18 (1.85–2.57)**	645	65%	**1.82 (1.51–2.18) **
Mode of HIV exposure						
MSM	971	33%	Reference	1202	52%	Reference
IDU	179	42%	**1.44 (1.15–1.81)**	160	57%	**1.41 (1.07–1.87) **
MSM/IDU	97	25%	**0.72 (0.56–0.92)**	119	41%	**0.67 (0.52–0.86) **
Heterosexual	284	44%	**1.92 (1.53–2.41)**	318	57%	**2.51 (1.90–3.32) **
Blood/pediatric	7	50%	2.23 (0.75–6.58)	7	64%	1.85 (0.56–6.12)
NIR	366	51%	**2.04 (1.67–2.48)**	437	71%	**2.62 (2.06–3.34) **
Residence at HIV diagnosis						
Inside King county	1049	34%	Reference	1298	53%	Reference
Outside King county	855	42%	**1.36 (1.20–1.53)**	945	61%	**1.37 (1.19–1.58) **
Country of origin						
US-born	1352	35%	Reference	1567	52%	Reference
Foreign-born	552	46%	**1.26 (1.06–1.51)**	676	67%	**1.34 (1.09–1.64)**

*Adjusting for gender, race/ethnicity, risk category, age at HIV diagnosis, residence in King County, and foreign-born status.

**Native Hawaiian or other Pacific Islander.

**Table 3:** Late HIV diagnoses by race/ethnicity and foreign-born status, Washington State, 2000–2009.

	Time-based late measure			Lab-based late measure		
	2000–2008			2000–2009		
	(Late = AIDS within 12 months of HIV)			(Late = initial CD4 < 350)		
	No.	% Late	Odds ratio (95% CI)*	No.	% Late	Odds ratio (95% CI)*
US-born						
White, NH	1011	34%	Reference	1190	51%	Reference
Black, NH	196	37%	1.07 (0.87–1.31)	203	55%	1.09 (0.86–1.38)
Hispanic	58	30%	0.98 (0.70–1.37)	83	57%	**1.51 (1.06–2.16) **
Asian	12	39%	1.59 (0.75–3.39)	15	54%	1.34 (0.62–2.88)
NHOPI**	3	30%	1.10 (0.27–4.42)	3	38%	0.69 (0.16–2.98)
AI/AN	43	49%	**1.67 (1.06–2.61)**	40	65%	1.68 (0.96–2.93)
Multiple/unknown	29	37%	1.35 (0.82–2.21)	33	58%	1.65 (0.95–2.86)
Foreign-born						
White, NH	66	31%	Reference	83	53%	Reference
Black, NH	186	49%	**2.16 (1.42–3.30)**	220	69%	**1.76 (1.08–2.88) **
Hispanic	215	48%	**2.33 (1.61–3.37)**	265	70%	**2.16 (1.43–3.27) **
Asian	68	46%	**2.03 (1.28–3.22)**	87	67%	**1.72 (1.03–2.88) **
NHOPI	8	43%	**3.41 (1.08–10.8)**	8	67%	2.11 (0.58–7.65)
AI/AN	2	33%	1.68 (0.29–9.85)	4	100%	n/a
Multiple/unknown	7	54%	3.13 (0.96–10.2)	9	75%	2.48 (0.61–10.1)

*Adjusting for gender, risk category, age at HIV diagnosis, and residence in King County.

**Native Hawaiian or other Pacific Islander.
